# HPLC method development/validation and skin diffusion study of caffeine, methyl paraben and butyl paraben as skin–diffusing model drugs

**DOI:** 10.1371/journal.pone.0247879

**Published:** 2021-03-17

**Authors:** Randa S. H. Mansour, Imad I. Hamdan, Mutaz S. H. Salem, Enam A. Khalil, ALSayed A. Sallam

**Affiliations:** 1 Faculty of Pharmacy, Philadelphia University, Amman, Jordan; 2 School of Pharmacy, University of Jordan, Amman, Jordan; 3 Al-Taqaddom Pharmaceutical Industries Inc., Amman, Jordan; Aristotle University of Thessaloniki, GREECE

## Abstract

The focus of this research was to develop and validate a suitable HPLC method, which allows simultaneous determination of three proposed skin model penetrants to investigate the percutaneous diffusion behavior of their combination: caffeine, methyl paraben and butyl paraben. These penetrants were selected because they represent a wide range of lipophilicities. This model highlights the effect of combining penetrants of different molecular properties on their diffusion behavior through skin. The proposed method employed a gradient system that was systematically optimized for separation and quantification of the penetrants. The effect of the stationary phase (C18, C4 and cyano (CN)) was assessed with CN proven to be superior in terms of peak shape, retentivity and dynamic linear range. Significant differences in retention time, peak broadening, and quantifiability between different stationary phases could be demonstrated. The method was validated as per ICH guidelines Q2 (R1) with a satisfactory outcome. The method was successfully applied for real diffusion experiments, and revealed notable differences between the individual penetrants and their ternary mixture on transdermal permeation. The method could potentially be extended to determine these analytes in other related skin permeation investigations.

## 1 Introduction

Transdermal drug delivery is the administration of a therapeutic agent through intact skin for systemic effect. It is considered as one of the most successful innovative research area in drug delivery [1] due to the numerous advantages it offers.

Previous investigations of the percutaneous permeation of various drugs (penetrants) have generally focused on the permeation of a single substance alone or combined with chemical penetration enhancers [2–5]. These approaches do not account for the possibility that the percutaneous permeation behavior of different penetrant substances combinations may alter the thermodynamic activity of specific penetrants or even affect the diffusion barrier properties of the stratum corneum (SC), leading to enhancing or retarding the diffusion of these penetrants through the skin [6, 7].

This research proposes a model, which consists of three model penetrants (caffeine (CAFF), methyl paraben (MP) and butyl paraben (BP)) chosen to represent molecules with a wide range of lipophilicities as dictated by their Log P values (Table 1) to investigate the diffusion behavior of their combination. The findings from our model can help to improve the understanding of the potential drug-drug [6, 7] or drug- excipient [8–10] combinations with transdermal penetration expected to be enhanced/retarded based on their lipophilicity. While the focus of this research is on the HPLC analytical method for the simultaneous determination of the proposed penetrants in *in-vitro* diffusion studies, the results of a comprehensive diffusion study will be published elsewhere.

**Table 1:** Structure and log P values of the proposed penetrants.

Compound	Log P	IUPAC Name	CAS Number
**CAFF**	-0.07 [11]	1,3,7-Trimethylpurine-2,6-dione	58-08-2
**MP**	1.93 [12]	Methyl 4-hydroxybenzoate	99-76-3
**BP**	3.57 [12]	Butyl 4-hydroxybenzoate	94-26-8

While some HPLC methods have been reported for simultaneous determination of parabens in pharmaceutical preparations [13, 14], only one method analyzed the most hydrophilic caffeine [15], and to the best of our knowledge, none was validated for percutaneous absorption. On the other hand, multiple published methods, including chromatographic, describe the determination of parabens in human urine, serum, tissue and breast milk, but exclude the hydrophilic caffeine. Such complex matrices dictated the use of extraction techniques and mostly performing the analysis using GC or LC coupled to a single or tandem mass spectrometer detector [16]. Meanwhile, it is well accepted that human skin is the best and most relevant type of skin for *in-vitro* diffusion testing [17]; thus, *in-vitro* permeation studies of human skin have their particular analytical considerations as usually no extraction step is involved, which increases the burden of the method’s required selectivity as some skin components may leak into the receiver compartment. Additionally, many skin diffusion experiments are performed using the more accessible UV detector rather than MS detectors, which also further stresses the need for a higher selectivity of the method.

Within the context of skin permeation research, the permeation of CAFF, MP and BP has been investigated as individual penetrants only; thus, a different analytical HPLC method for each penetrant was developed [18, 19]. In a recent study, the determination of methyl, propyl, and butyl parabens, but not CAFF, as individual skin penetrants was accomplished using the same method that utilized LC-MS/MS [20].

Therefore, there is a need for simple and reliable chromatographic method that enables the separation and quantification of the chosen model drugs (CAFF, BP and MP) in the settings of human skin diffusion studies. The aim of the present study was to develop and validate a simultaneous HPLC/ UV detection method of the three model penetrants comprising the hydrophilic and lipophilic models of drugs. Furthermore, the simultaneous determination of the proposed penetrants was investigated to characterize their percutaneous permeation when combined in transdermal drug delivery systems. To the best of our knowledge, there has been no published method for the selected model drugs serving that purpose. Moreover, unlike our proposed method, previous studies involved in skin permeation had focused on the pharmaceutical aspect of the research without a full description of the development and validation of the analytical aspects. Finally, the proposed model of penetrants, coupled with the proposed analytical method, is applicable in various investigations related to *in-vitro* diffusion studies and there is a high potential to extend the analytical method for studies that utilize diffusion barriers other than SC, such as skin dermatomes and various biological membranes.

## 2 Methods

### 2.1 Materials and equipment

CAFF (anhydrous) was obtained from BASF, Ludwigshafen, Germany. MP and BP were obtained from Sigma Chemicals Co., Gillingham, UK. Isopropyl myristate (IPM), trypsin (from porcine pancreas, lyophilized powder, 1000–2000 BAEE units/mg solid) and cellulose membrane (MWCO 12000 Da) were purchased from Sigma-Aldrich, St. Louis, Missouri, USA. Acetonitrile HPLC grade was purchased from EMD Millipore, Darmstadt, Germany. Hydroxypropylmethylcellulose (HPMC) was obtained from Dow Chemical Company, Midland, Michigan, USA.

Columns examined included cyano (CN), C18 columns (Phenomenex^®^, Torrance, CA, USA) in addition to C4 column (Sepax Bio, Newark, Delaware, USA). All columns examined had the same dimensions (4.6x150 mm, 5 μm). Other specifications for CN column were: 100 Ǻ pore diameter, 400 m^2^/g surface area and 7% carbon load. The pore sizes of the C18 and C4 column were 100 and 300 Ǻ respectively. In all cases, the columns were connected to a security guard cartridge (4x3 mm) from Phenomenex^®^, Torrance, CA, USA.

Shimadzu LC-2010A HT HPLC system (Kyoto, Japan) equipped with an autosampler, degasser, column temperature controller and UV-VIS detector with a built-in mercury lamp and standard temperature-controlled cell was employed for method development, validation and analysis of real samples. The system was also equipped with LC solution software, which was used for data analysis and reporting. *In-vitro* diffusion studies were performed using PermeGear jacketed Franz diffusion cells (Hellertown, PA, USA). Ultra-Turrax homogenizer (Janke and Kunkel Ika-Turrax, Mönchengladbach, Germany) was used to prepare the emulgel formulations.

### 2.2 General procedures

#### 2.2.1 Preparation of human skin sheets

The study was approved by the Ethics Committee for Scientific Research on Humans at Philadelphia University. The approval of the skin donor, a 40-year-old female: was verbally granted directly, since she owned the samples after conducting abdominal plastic surgery and had the option to bury them or donate to scientific research. Her approval was granted at two different occasions, before and after conducting the surgery in the presence of her surgeon.

The skin was obtained from a local hospital immediately after the surgery, and immediately defatted using a scalpel, cleaned by tapping with dry wipes and neatly placed on paperboard wrapped with aluminum foil so that the skin surface is facing upward. The skin was then covered with aluminum foil, kept in zipper plastic bags and subsequently stored at -70°C for a maximum period of 6 months.

#### 2.2.2 Isolation of stratum corneum (SC)

Punched skin discs of 25mm diameter were placed in a petri dish with SC side up, and 1%w/v trypsin was added until the skin surface was immersed. The petri dish was then covered and incubated at 32°C for 24–48 h until the separation of the SC was evident. The separated SC sheets were rinsed several times with distilled water.

### 2.3 *In-vitro* diffusion studies

#### 2.3.1 Preparation of the drug loaded emulgel formulations

Emulgels containing 15% of IPM and 2% of the gelling agent, HPMC, were prepared. IPM was added to a portion of water, and the mixture was homogenized by a hand homogenizer for a minute at 8000 r.p.m. The system was then jellified by the addition of HPMC followed by homogenization for 5 min at 8000 r.p.m. to stabilize the emulsion. The emulgels were loaded with infinite doses of the corresponding model penetrant (CAFF, MP or BP) and then left for 24 h to ensure the absence of air bubbles and formation of a suspension (presence of insoluble drug particles). Emulgel containing a mixture of the three drugs (ternary mixture) was also prepared using the same method. Analyte free-emulgel was prepared following the same procedure without the addition of the model penetrants.

#### 2.3.2 Performing the *In-vitro* diffusion studies

*In-vitro* diffusion studies were performed using stirred Franz diffusion cells of 1cm^2^ diffusion area. The cells were jacketed with water at 32°C. Skin disks of 25 mm in diameter were punched out and thawed; the SC sheets were separated and washed as described in section 2.2.2, then gently pressed by a filter paper and soaked in phosphate buffer pH 6.8 for 30 min before being ready to be mounted on the diffusion cells. The receiver compartment (8 mL) was filled with phosphate buffer pH 6.8 previously filtered by 0.45μm nylon filter and equilibrated at 32°C. After visually checking its integrity, a SC sheet was placed on a piece of cellulose membrane previously soaked in phosphate buffer pH 6.8 and then mounted on the top of the receiver compartment. The cellulose membrane serves as a support for the SC sheet. Extreme care was provided not to entrap any air bubbles under the membrane or between the membrane and the SC. The donor compartment was then placed and fastened by a clamp. Adjustment of the receiver volume was made followed by placing 1g of the corresponding drug-containing emulgel or ternary mixture emulgel. The emulgel preparations were free of air bubbles, and care was taken to not entrap any air bubbles between the formulation and the SC. The donor was then completely covered by parafilm, and the cells were stirred at 32°C for 24–26 h. At pre-specified time intervals (0.25, 0.5, 1, 2, 3, 5, 7, 12 and 24–26 h), 500μL of the receiver buffer was withdrawn and immediately replaced with an equal volume of fresh buffer previously filtered by 0.45μm nylon filter and equilibrated at 32°C. The experiments were performed as 4–6 replicates. At the end of each diffusion experiment, the emulgels were inspected to confirm the presence of suspended drug particles. The withdrawn samples were analyzed using the final proposed chromatographic conditions shown in the subsequent section (2.5). Appropriate dilutions were made whenever required. The diffusion profiles were constructed by plotting the average cumulative diffused amount (μg/ml/cm^2^) versus time (h). Refer to Tables 1A, 2A and 3A in S1 Appendix for data used in the construction of the diffusion profiles.

### 2.4 Preparation of standards and quality control solutions

A stock solution of each analyte was prepared in 40% methanol in phosphate buffer pH 6.8. The stock solution concentration was 800 μg/mL for CAFF and 400 μg/mL for MP and BP. Proper dilutions were made to prepare a solution for each individual analyte, CAFF, MP or BP having a 25μg/ml concentration. A mixture of all analytes containing 25μg/ml of each was also prepared. The prepared individual and mixture solutions were used for method development.

From each analyte’s stock solution, proper dilutions were made to prepare a series of solutions of CAFF, MP or BP. CAFF solutions were 0.02, 0.2, 0.4, 0.8, 2, 4, 12, 20, 28, 36 and 40 μg/mL whereas BP solutions were 0.02, 0.2, 0.4, 0.8, 2, 4, 12, 28, 36 and 40 μg/mL. MP solutions were 0.8, 2, 4, 12, 28, 36 μg/mL. In all cases, three replicates of each solution were prepared and used for linearity testing.

Using analyte-free emulgel in the donor compartment with phosphate buffer pH 6.8 as the receiver and SC as the diffusion barrier, diffusion experiment, as in section 2.3.2, was conducted in six replicates for 26 h. The analyte-free emulgel was prepared as in section 2.3.1. The collected buffer solutions from the receiver compartments were pooled and used as the matrix buffer for selectivity testing. The matrix buffer was also used to prepare quality control (QC) solutions for precision and accuracy testing by diluting the relevant stock solution. For each analyte, three QC solutions were prepared in triplicate: low (0.8 μg/mL), medium (16 μg/mL) and high (32 μg/mL).

### 2.5 Instrumentation and analytical conditions

The final optimized chromatographic conditions for the separation and quantification of the mixture (CAFF, MP and BP) consisted of a cyano column (4.6x150 mm, 5 μm, 100 Ǻ pore diameter, 400 m^2^/g surface area and 7% carbon load, Phenomenex^®^, USA) connected to a 4x3 mm security guard cartridge (Phenomenex). The temperature of the column was maintained at 25 ˚C, and the flow rate was kept at 1mL/min, with a detection wavelength set at 268nm and an injection volume of 100μL. A gradient mobile phase based on acetonitrile (solvent B) and water (solvent A) was employed and can be detailed as: 5% B to 50% B within 10min, 50% B to 90% B within 5min, 90% B to 5% B within 1min, and kept at 5% B for 4min.

### 2.6 Method development

To examine the retention of CAFF, MP and BP, the prepared individual analyte solutions were separately injected into three columns (CN, C18 and C4) using a series of mobile phases containing increasing percentages of acetonitrile in water (v/v%) by isocratic elution. The injections were performed in triplicate.

The mixture of the analytes was tested in CN and C18 columns. Different gradient systems of acetonitrile and water were examined in an effort to optimize the separation of the mixture components. The injections were performed in triplicate.

Three volumes of injection of the mixture (25, 50 and 100μL) were attempted on CN and C18 columns using the final gradient system, which was found to provide a satisfactory separation of CAFF, MP and BP. The injections were performed in triplicate.

### 2.7 Method validation

#### 2.7.1 Selectivity (specificity)

The matrix buffer prepared in section 2.4 was injected using the final chromatographic conditions. Selectivity was assessed based on the presence or absence of co-eluting peaks at the analytes’ retention times.

#### 2.7.2 Linearity

The prepared linearity solutions (section 2.4) were analyzed according to the final chromatographic conditions. The calibration curve for each analyte was constructed by plotting the average peak area (of three determinations) versus the corresponding concentration. Refer to Tables 1A, 2A and 3A in S1 Appendix for data used in the construction of the calibration curves.

#### 2.7.3 Accuracy

Each QC solution (section 2.4) was analyzed according to the final chromatographic conditions. The procedure was repeated on two different days. The calculated concentration for each injection was obtained from the calibration curve equation, and the corresponding % recovery was calculated according to the following equation:

$$\%Recovery=\frac{Calculated\;Concentration}{Theoritical\;Concentration}\times 100$$ Eq 1

The % recovery value for each analyte for each of the QC solutions, intra- and inter-day, was expressed as mean recovery with the corresponding %RSD value. Additionally, the corresponding mean concentration ± SD was also reported.

#### 2.7.4 Precision

Precision was evaluated using the QC samples described in section 2.4. For each analyte, repeatability (intra-day precision) was assessed by analyzing each QC solution according to the final chromatographic conditions. The intermediate precision (inter-day precision) was evaluated by repeating the procedure on two different days. In both cases, the % RSD of the % recovery for all QC solutions was obtained and reported.

#### 2.7.5 Limit of quantitation (LOQ) and limit of detection (LOD)

For each analyte, proper dilutions of the linearity solution with the lowest concentration in section 2.4 were prepared and analyzed according to the final chromatographic conditions. Concentrations that provided a signal-to-noise ratio > 3 and > 10 were taken as LOD and LOQ, respectively.

## 3 Results and discussion

### 3.1 Method development

The retention behavior of the model drugs on three different types of commonly employed stationary phases was investigated. The main challenge in separating our mixture of model drugs was the components having vast differences in lipophilicity, which most likely would require a gradient system. However, we evaluated the retentivity of the various analytes (based on the capacity factor (K) values) under isocratic conditions. The peak shape was also considered in deciding the best performance.

The value of K was calculated according to Eq 2, and for each analyte, the obtained log K values on each column were plotted against the percentage of acetonitrile.

$$K=\left(\frac{t_{r}-t_{0}}{t_{0}}\right)100$$ Eq 2

Where *t_r_* is the corresponding analyte retention time (min), and *t*_0_ is the unretained species retention time (solvent front peak, min).

**Fig 1:**
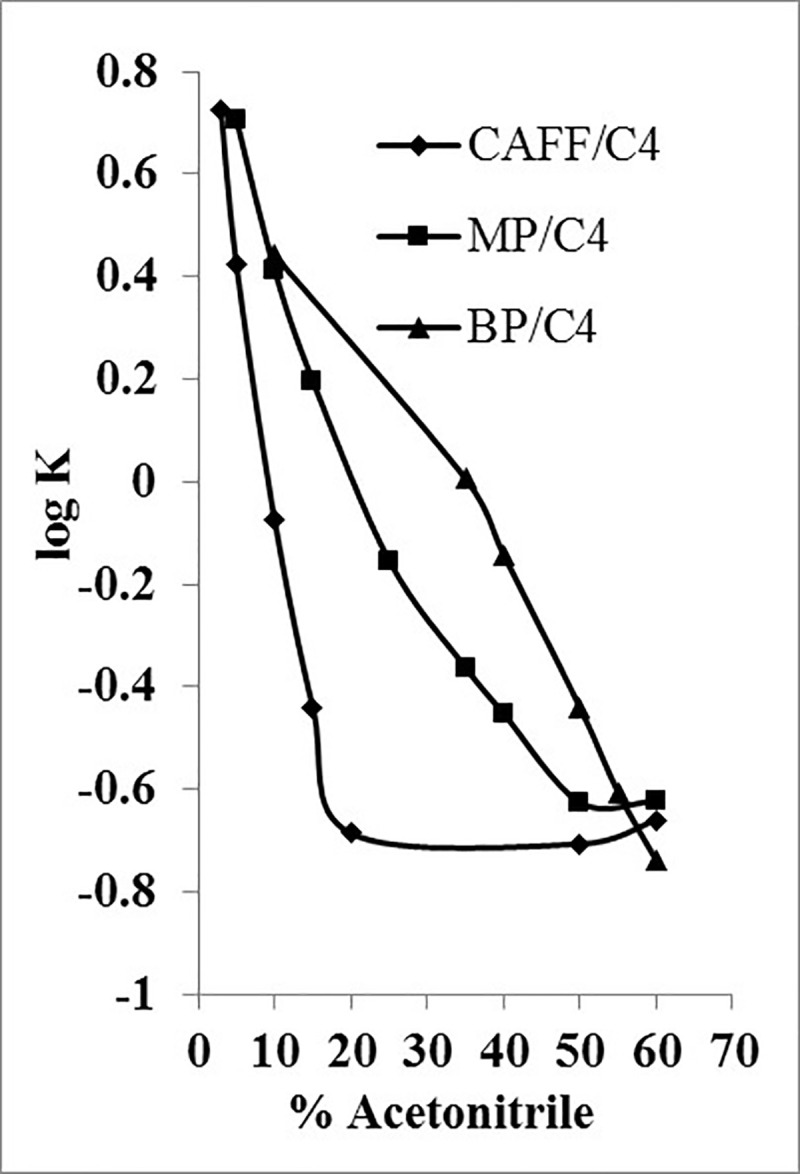
log K of CAFF, MP and BP as a function of acetonitrile concentration using C4 column.

Using C4 stationary phase, a typical reversed-phase mechanism appeared to occur because the retention increased in parallel with the increase in hydrophobicity of the analyte (BP ˃ MP ˃ CAFF), as shown in Fig 1. Mobile phases with percentages of acetonitrile ˃ 40% appeared to be strong for the analytes to the point that they all eluted at almost the same t_r_ < 1min. While separation of all analytes could be achieved employing the isocratic percentages in the range of approximately 15–30%, CAFF did not show satisfactory retention that would ensure the absence of interferences. At lower percentages of acetonitrile, t_r_ of the more lipophilic analytes started to become significantly longer with broader peaks, i.e., lower efficiency with minimal resolution. Therefore, C4 stationary phase was excluded from further development.

**Fig 2:**
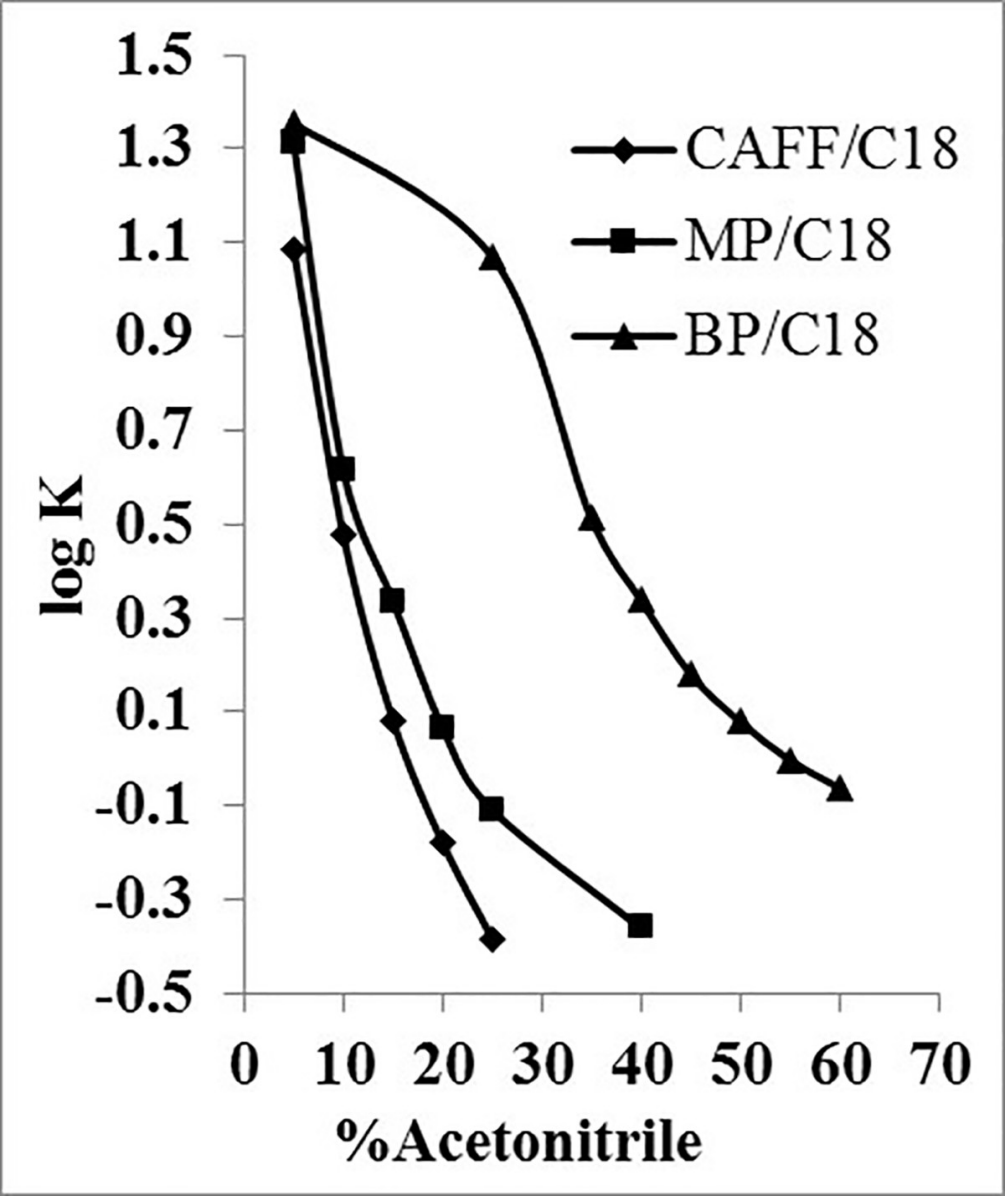
log K of CAFF, MP and BP as a function of acetonitrile concentration using C18 column.

Using C18 column, almost a typical (near-linear) plot of log K versus percentage of organic solvent was obtained (Fig 2). However, no selectivity differences could be obtained between CAFF and MP, the least and the intermediate hydrophobic analytes respectively, except at low percentages of acetonitrile (< 10%). Accordingly, the retention times for MP and BP would be impractically long (> 20 min). Thus, C18 appeared to provide very low retention for CAFF and very high retention for BP, which is not ideal for practical separation of the mixture even if gradient elution was considered. Nevertheless, C18 was considered for further method development.

**Fig 3:**
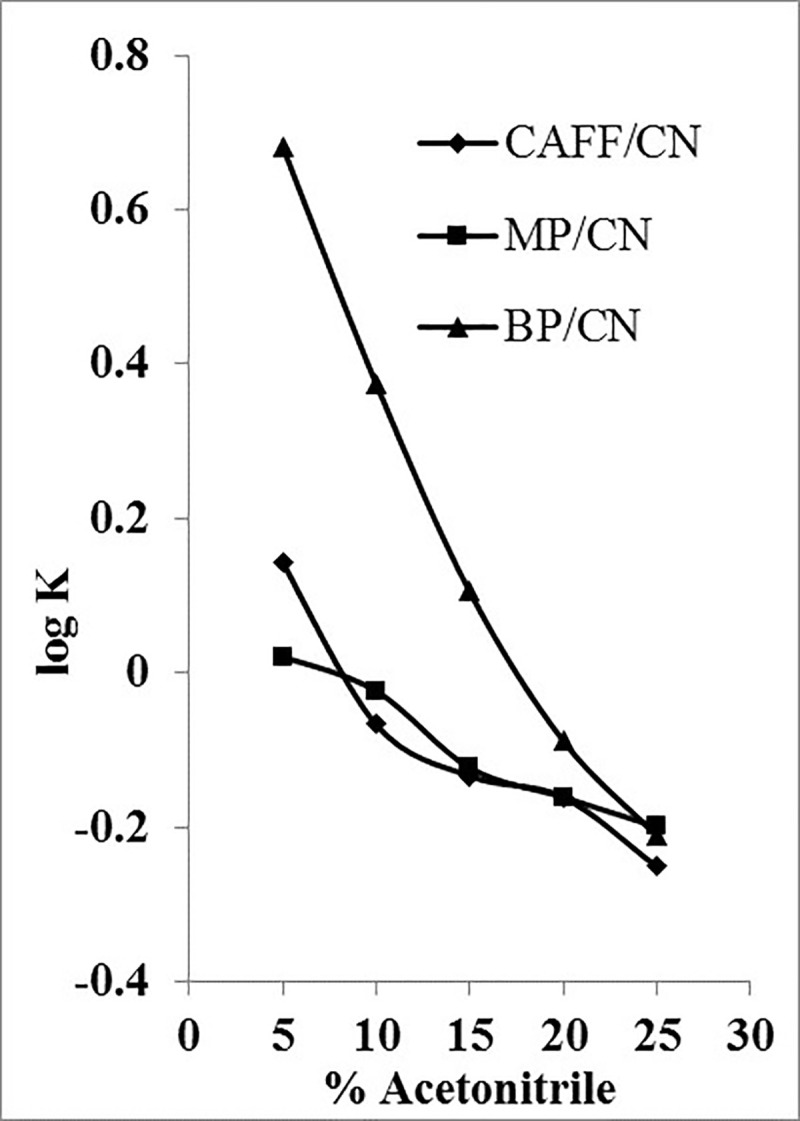
log k of CAFF, MP and BP as a function of acetonitrile concentration using CN column.

Cyano stationary phases are rather hydrophilic and could provide a different selectivity of our group of analytes; thus, their performance was assessed. Fig 3 shows the plots of log K versus the percentage of acetonitrile for CN column. While potential isocratic separation could only be achieved at lower percentages of acetonitrile in a similar manner to C18 column, CN stationary phase appeared to better suit the components of the mixture because the time window between the least and most retained analytes was < 5 min. Therefore, CN phase appeared to perform better than C18, particularly as the analytes’ peak shapes were also superior.

It is noteworthy that the order of elution of CAFF and MP on CN column was dependent on the percentage of acetonitrile in the mobile phase (Fig 3). This suggests that for the more hydrophilic compounds (CAFF and MP), a dual reversed and normal phase mechanism of retention might occur. At low percentages of acetonitrile (< 10%), normal phase retention for CAFF and MP was predominant; thus, causing the elution of CAFF after MP. It is also evident that this phenomenon increased the column efficiency for the separation of CAFF and MP and can be employed in the gradient elution to optimize the separation of these two potentially overlapping analytes. Dual mechanisms of retention on reversed-phase columns have been reported previously [21].

**Fig 4:**
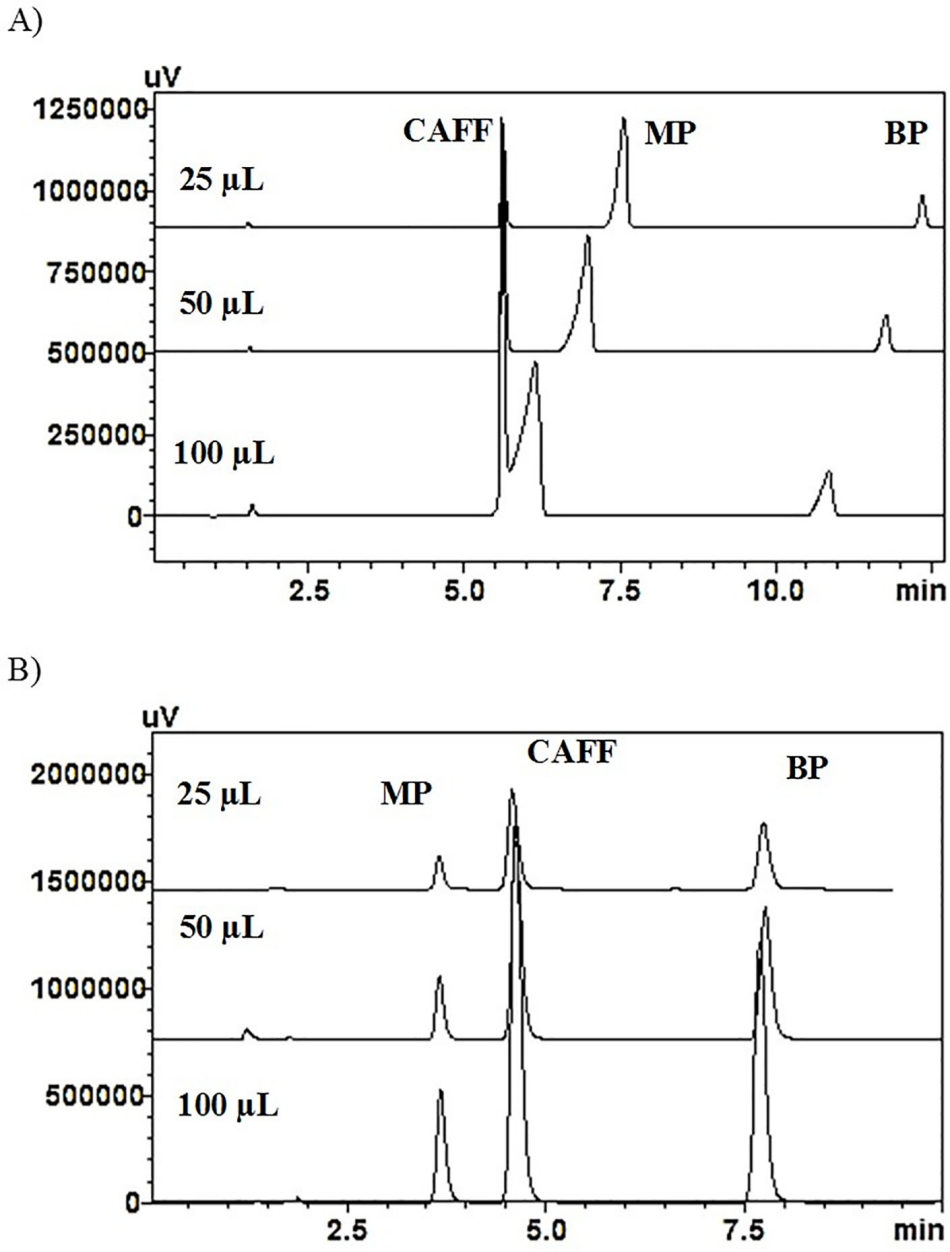
Elution of the analytes mixture at different injection volumes (25, 50 and 100μL) using A) C18 and B) CN columns.

As gradient elution was concluded to be essential, different gradient systems were tested (using both stationary phases C18 and CN). Attempts to maximize the separation of CAFF and MP started with a low percentage of acetonitrile, which was then slowly increased over time, leading to elution of BP within a reasonable retention time. The final gradient system that was adopted for further testing of both columns is shown in section 2.4. Interestingly, CAFF and MP exhibited different elution orders on CN and C18 columns (Fig 4).

In diffusion studies, the amounts of the penetrants diffused initially are usually low, especially for penetrants of a low and high degree of hydrophobicity such as CAFF and BP, respectively. This may limit their quantification at the first period of the diffusion study. It is crucial to quantify the amounts diffused during the first period to evaluate certain essential diffusion parameters, such as the lag time. This limitation can be minimized by increasing the injection volume in the analysis thus, it was decided to increase the injection volume as much as possible, i.e., 100μL.

Fig 4 shows the effect of increasing the injection volume of the analyte mixture on their separation using C18 and CN columns. It is obvious that C18 column exhibited an amount-dependent elution pattern, particularly for MP. MP showed a significant shift in t_r_ and an increase of peak broadening as the injected volume was increased. At an injection volume of 100μL, the overlap between MP and CAFF was serious and prohibiting the quantitative use of the method. On the other hand, the performance of CN column was not affected by the amount of any of the analytes, and showed a steady performance at the investigated level. As a result, C18 column was excluded from further method development at this stage.

The performance of CN column was found to be superior to the other tested stationary phases for this particular application, i.e., *in-vitro* diffusion studies. Thus, it was selected as the column of choice for method validation.

### 3.2 Method validation

The method was validated in accordance with ICH guidelines [22] in terms of selectivity, linearity range, accuracy, precision, LOQ and LOD.

#### 3.2.1 Selectivity (specificity)

During the course of the diffusion experiment, substances other than the analyte are expected to co-present in the receiver compartment of the diffusion cell. These substances include the buffer and SC constituents that may leach and diffuse to the receiver compartment. In principle, such a substance might co-elute with any of the analytes and constitute a major interference. The absence of major interfering UV-absorbing species at the same t_r_ of the corresponding analyte was confirmed by visual inspection of the chromatograms. If any peaks were observed, the percentage interference was calculated according to the following equation

$$\%Interference=\left(\frac{{PA}_{interference}}{{PA}_{LL}}\right)100$$ Eq 3

Where PA_interference_ is the average peak area of the observed peak (n = 6), *PA*_*LL*_ is the average analyte peak area of the lower level concentration of the linear range for the corresponding analyte (n = 6). Usually, the interference is calculated based on the peak area of the target concentration, but this was not applicable in our case. Consequently, the worst-case scenario was considered, i.e: the obtained peak area of the potential interfering peak from the buffer matrix (26 h-incubated SC buffer) was compared to the peak area of the lower level concentration that represents samples withdrawn at the early stages of the diffusion experiments.

**Fig 5:**
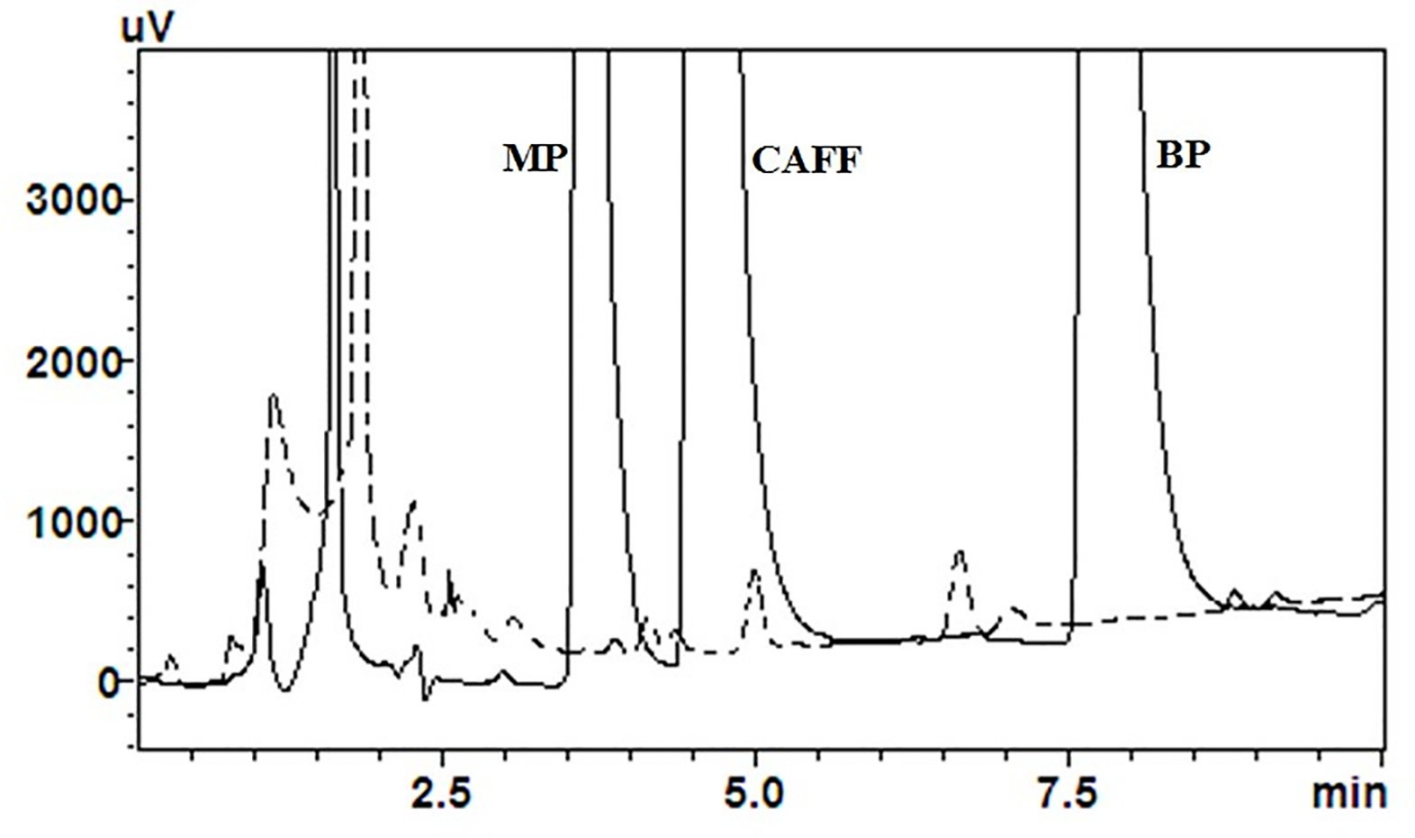
Comparison between the chromatogram for the mixture of analytes (continuous line) and that of the matrix (dotted line). The concentration of the mixture is 25 μg/mL with respect to each analyte.

Upon comparing the chromatogram for the mixture of analytes with that of the matrix, interfering peaks with MP and CAFF were evident (Fig 5). Calculated based on Eq 3, the percentages of interference with MP and CAFF peaks were only 3.2% and 1.9%, respectively. At higher concentration levels (16 μg/ml, the intermediate level QC concentration), the estimated percentages of interferences were even smaller, i.e., 0.18% and 0.003% for MP and CAFF, respectively. Thus, the proposed chromatographic conditions were concluded to be selective enough for the intended purpose.

The simultaneous chromatographic determination of CAFF, MP and BP in soft drinks was previously described, among other ingredients [15]. The proposed method was selective for its intended application not for *in-vitro* diffusion studies involving human SC.

#### 3.2.2 Range

The ICH guidelines dictate setting the range of the analytical method based on the target concentrations intended for analysis; thus, minimum specifications for the lower and upper limits are required for the assay of an active substance or finished product, content uniformity, dissolution testing and determination of an impurity [22]. For the current intended (or related) application of the proposed method, there is no previous prediction of the target concentrations. As a result, the range was derived from the linearity studies taking into consideration widening the range as long as linearity, accuracy and precision are met. Emphasis was made on the lower limit to allow the analysis of samples withdrawn at the early stages of the diffusion studies, particularly for the slowly diffusing analytes, CAFF and BP. The obtained ranges for the analytes are represented in Table 2.

**Table 2:** Linearity ranges, equations, r^2^ values and number of concentrations of the calibration curves for CAFF, MP and BP.

	Linearity range (μg/mL)	Equation	r^2^	Number of concentrations
**CAFF**	0.02–40	y = 257526x +3930.6	0.9988	11
**MP**	0.8–36	y = 446383x + 5262	0.9997	6
**BP**	0.02–40	y = 246384x +1138.3	0.9996	10

y is the peak area, and x is the concentration in μg/mL

n = 3

The obtained ranges demonstrated acceptable lower ranges that were low enough to permit the analysis of the early-withdrawn samples. At the same time, the upper limits were practically reasonable to minimize the number of samples withdrawn toward the end of the diffusion experiments and required dilution. A chromatographic method reported for simultaneous determination of parabens in sunscreens demonstrated a relatively high lower limit of obtained ranges (10–200 μg/mL), making it not practical to be applied for the current purpose of analysis [13]. In another method utilized for the analysis of CAFF and parabens in soft drinks, the obtained ranges were very narrow (32–48 ppm) to be practically applied for the current application [15]. One study employed HPLC analysis of CAFF, MP and BP in *in-vitro* skin permeation within a practical range of 0.05–20 μg/mL; however, the permeants were analyzed individually [20].

#### 3.2.3 Linearity

The calibration curves based on the prepared series of concentrations for each analyte were constructed. At all concentrations, the % RSD value was below 2. Generally, a regression coefficient (r^2^) > 0.998 is considered as evidence of an acceptable fit of the data to the regression line [23]. The obtained data presented in Table 2 indicates that linearity was demonstrated for all the analytes in their corresponding ranges of concentrations.

#### 3.2.4 Precision and accuracy

To establish precision, repeatability and intermediate precision were evaluated by calculating the % RSD of the %recovery values for all QC concentrations within the same day and across different days. In addition, accuracy was evaluated by calculating the average % recovery for each set of QC solutions. Table 3 summarizes the obtained data. In all cases, the RSD values were not more than 2%. The average % recovery was within the limits of +/- 4% of the target amount except for BP (up to + 9%. Nonetheless, the method was considered sufficiently precise and accurate.

**Table 3:** Data of accuracy and precision for CAFF, MP and BP.

				CAFF	MP	BP
**Precision Mean %RSD**	**n = 9**		**Intra-day**	0.89	1.08	1.84
	**n = 18**		**Inter-day**	2.00	1.57	1.93
**Accuracy (n = 3 for intra-day and 6 for inter-day)**	**Lowest 0.8 μg/mL**	**Day 1**	**Mean Concentration±SD**	0.797±0.002	0.801±0.001	0.852±0.004
			**Mean %Recovery**	99.7	100.1	106.5
			**%RSD**	0.19	0.18	0.48
		**Day 2**	**Mean Concentration±SD**	0.824±0.004	0.781±0.003	0.857±0.007
			**Mean %Recovery**	102.9	97.7	107.1
			**%RSD**	0.46	0.32	0.78
		**Inter-day**	**Mean Concentration±SD**	0.811±0.015	0.791±0.011	0.854±0.006
			**Mean %Recovery**	101.3	98.9	106.8
			**%RSD**	1.79	1.36	0.66
	**Intermediate 16 μg/mL**	**Day 1**	**Mean Concentration±SD**	15.626±0.002	15.967±0.017	17.338±0.004
			**Mean %Recovery**	97.7	99.8	108.4
			**%RSD**	0.01	0.11	0.02
		**Day 2**	**Mean Concentration±SD**	15.513±0.015	16.263±0.039	17.370±0.094
			**Mean %Recovery**	97.0	101.6	108.6
			**%RSD**	0.10	0.24	0.54
		**Inter-day**	**Mean Concentration±SD**	15.569±0.063	16.115±0.164	17.354±0.062
			**Mean %Recovery**	97.3	100.7	108.5
			**%RSD**	0.41	1.02	0.36
	**Highest 32 μg/mL**	**Day 1**	**Mean Concentration±SD**	31.532±0.022	32.660±0.079	33.245±0.030
			**Mean %Recovery**	98.5	102.1	103.9
			**%RSD**	0.07	0.24	0.09
		**Day 2**	**Mean Concentration±SD**	31.507±0.015	32.552±0.006	33.181±0.273
			**Mean %Recovery**	98.5	101.7	103.7
			**%RSD**	0.05	0.02	0.82
		**Inter-day**	**Mean Concentration±SD**	31.519±0.022	32.606±0.078	33.213±0.177
			**Mean %Recovery**	98.5	101.9	103.8
			**%RSD**	0.07	0.24	0.53

#### 3.2.5 LOQ and LOD

The LOQ and LOD values of each analyte were considered as the concentrations with S/N ratios of ˃10 and ˃3, respectively (Table 4). The obtained values are sufficiently low to enable accurate determination of diffused analytes at low concentrations within the early stage of diffusion experiment. A study described the analysis of parabens in *in-vitro* diffusion experiments demonstrated LOD values of less than 1 ng/mL. The study employed LC-MS/MS analysis for the individual, not the simultaneous analysis of parabens, and CAFF was not included [20].

**Table 4:** Data of LOQ and LOD (μg/mL) for CAFF, MP and BP (n = 3).

	LOQ	LOD
**CAFF**	0.005	0.0025
**MP**	0.001	0.0005
**BP**	0.02	0.01

### 3.3 Application of the proposed method

**Fig 6:**
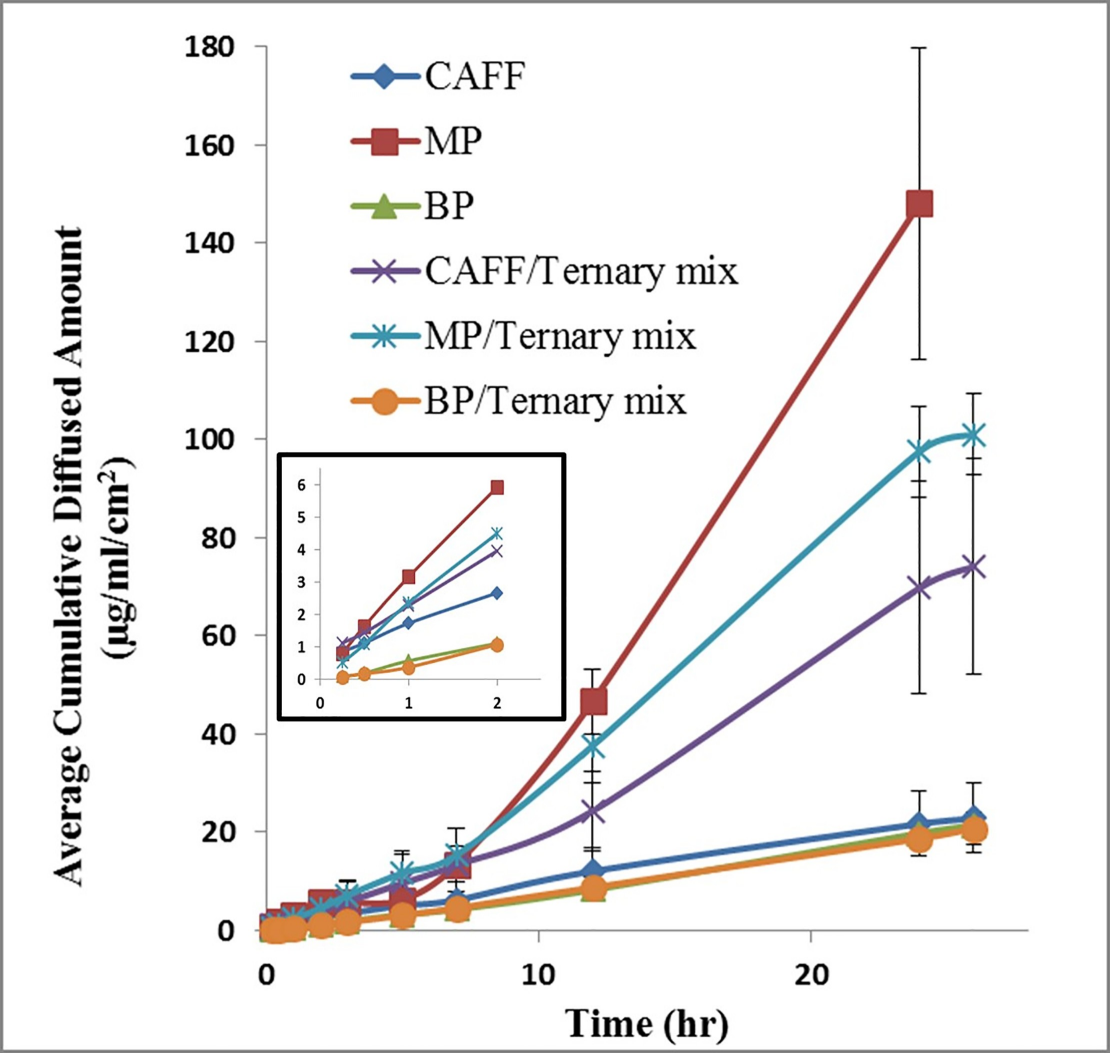
Diffusion profiles, through stratum corneum, of CAFF, MP and BP from their corresponding individual and ternary mixture emulgels. The plot in the insert shows the points obtained at the early stage of the experiment. n = 4–6.

To illustrate the applicability of the described method, *in-vitro* diffusion studies of the proposed individual penetrants and their ternary mixture were performed, using human SC and emulgel formulations. The samples withdrawn from the receiver media were analyzed according to the final chromatographic conditions described above. The resulting diffusion profiles were constructed and shown in Fig 6.

The analytical method was successfully used to quantify the diffusion of the individual as well as combined model penetrants through human SC. The diffused amounts at the early stages of the experiments were successfully quantified. This is particularly crucial for calculating diffusion-related parameters such as the lag time.

The effect of the penetrants combination on their permeation behavior is evident, particularly the retardation of MP and enhancement of CAFF permeation. These observations suggest some interaction between the two components (CAFF and MP). Such interaction could occur either in the donor vehicle or as the penetrants traveled through SC leading to improvement in the diffusion of CAFF and a decrease in the diffusion of MP. The suggested potential interaction requires further explorations and further experiments are planned to be conducted to investigate these effects and will be published somewhere else.

This study showed that in the presence of binary or ternary penetrants (drugs/excipients) combinations, the percutaneous diffusion behavior would be different from that of a single penetrant due to the interaction between the penetrants themselves or their interaction with the SC, which affect their permeability or partitioning performance. Regardless of the nature of the potential interaction and the involved mechanism of retardation/enhancement effect, the possibility of this could not have been revealed if only individual diffusion experiments were performed. Therefore, our findings further emphasize the need to perform diffusion experiments in the simultaneous presence of the potential penetrants to investigate potential interaction during the diffusion process itself.

## 4 Conclusion

As per ICH guidelines Q2 (R1), a validated HPLC method was systematically optimized for the analysis of the samples obtained from the diffusion studies of three penetrants (CAFF, MP and BP). The model penetrants are of different lipophilicity, and can be adopted, along with the proposed analytical method, for various investigations related to *in-vitro* percutaneous permeation, such as quantification of the effect of formulation variables (e.g., potential penetration enhancers) and process variables (e.g., various conditions of the diffusion study). The method is considered relatively simple since it is based on acetonitrile/water gradient elution. There is also a high potential to extend the analytical method for studies that utilize diffusion barriers other than SC, such as skin dermatomes and biological membranes. In addition, the method allows the simultaneous determination of the penetrants, which permits conducting the diffusion studies on individual penetrants as well as binary and ternary mixtures of them. The performance of CN column was found to be superior to C18 and C4 columns in terms of peak shape, retentivity and linear range. Additionally, the method demonstrated acceptable validation parameters and application of the method to *in-vitro* diffusion studies of the proposed individual penetrants and their ternary mixture was successfully demonstrated. Our findings highlight the need for investigating the permeation behavior of a penetrant of interest in the presence of other penetrating substances and simultaneous determination of these penetrants is necessary to obtain reliable results from such investigations.

## Supporting information

S1 Appendix *In-vitro* permeation study. (DOCX)

S2 Appendix Data of linearity. (DOCX)
