# Supplementary material for: HPLC method development/validation and skin diffusion study of caffeine, methyl paraben and butyl paraben as skin–diffusing model drugs
Source: PLoS One. 2021 Mar 17;16(3):e0247879. doi: 10.1371/journal.pone.0247879 (PMC7968716; doi:10.1371/journal.pone.0247879)
Supplement: S1 Appendix — (DOCX) [file pone.0247879.s001.docx]

**S1 Appendix**

**In-vitro permeation study**

**Table 1A: Cumulative diffused amount (µg/ml/cm^2^) for caffeine**

|  | **Caffeine alone** | | **Caffeine in ternary mix** | |
| --- | --- | --- | --- | --- |
| **Time (h)** | **Average** | **SD** | **Average** | **SD** |
| **0.15** | 0.86 | 0.32 | 1.13 | 0.81 |
| **0.5** | 1.12 | 0.33 | 1.44 | 1.99 |
| **1** | 1.73 | 0.23 | 2.28 | 2.56 |
| **2** | 2.67 | 0.50 | 3.96 | 3.44 |
| **3** | 3.49 | 0.77 | 5.84 | 4.30 |
| **5** | 5.06 | 1.22 | 9.59 | 5.87 |
| **7** | 6.20 | 1.56 | 13.32 | 7.32 |
| **12** | 12.07 | 4.64 | 24.35 | 8.14 |
| **24** | 21.72 | 6.67 | 69.73 | 21.64 |
| **26** | 22.82 | 7.09 | 74.11 | 21.98 |

**Table 2A: Cumulative diffused amount (µg/ml/cm^2^) for methyl paraben**

|  | **Methyl paraben alone** | | **Methyl paraben in ternary mix** | |
| --- | --- | --- | --- | --- |
| **Time (h)** | **Average** | **SD** | **Average** | **SD** |
| **0.15** | 0.81 | 0.34 | 0.53 | 0.61 |
| **0.5** | 1.65 | 0.36 | 1.09 | 0.86 |
| **1** | 3.16 | 0.41 | 2.37 | 1.22 |
| **2** | 5.92 | 0.63 | 4.50 | 2.61 |
| **3** | 5.73 | 1.66 | 7.15 | 2.83 |
| **5** | 6.22 | 3.32 | 11.69 | 4.37 |
| **7** | 13.51 | 3.53 | 15.34 | 5.61 |
| **12** | 46.69 | 6.66 | 37.64 | 7.70 |
| **24** | 148.07 | 31.68 | 97.55 | 9.30 |
| **26** | NA | NA | 101.04 | 8.28 |

**Table 3A: Cumulative diffused amount (µg/ml/cm^2^) for butyl paraben**

|  | **Butyl paraben alone** | | **Butyl paraben in ternary mix** | |
| --- | --- | --- | --- | --- |
| **Time (h)** | **Average** | **SD** | **Average** | **SD** |
| **0.15** | 0.10 | 0.04 | 0.08 | 0.02 |
| **0.5** | 0.20 | 0.09 | 0.18 | 0.06 |
| **1** | 0.57 | 0.15 | 0.38 | 0.24 |
| **2** | 1.11 | 0.36 | 1.08 | 0.29 |
| **3** | 1.87 | 0.32 | 1.70 | 0.43 |
| **5** | 3.24 | 0.58 | 3.07 | 0.79 |
| **7** | 4.31 | 0.77 | 4.57 | 1.11 |
| **12** | 8.27 | 1.59 | 8.77 | 2.22 |
| **24** | 19.80 | 2.55 | 18.70 | 3.63 |
| **26** | 21.40 | 2.68 | 20.76 | 3.36 |
