# Supplementary material for: HPLC method development/validation and skin diffusion study of caffeine, methyl paraben and butyl paraben as skin–diffusing model drugs
Source: PLoS One. 2021 Mar 17;16(3):e0247879. doi: 10.1371/journal.pone.0247879 (PMC7968716; doi:10.1371/journal.pone.0247879)
Supplement: S2 Appendix — (DOCX) [file pone.0247879.s002.docx]

**S2 Appendix**

**Data of linearity**

**Table 1B: Linearity data for caffeine**

|  |  | **Standard concentration (µg/mL)** | | | | | | | | | |
| --- | --- | --- | --- | --- | --- | --- | --- | --- | --- | --- | --- |
|  | **0.02** | **0.2** | **0.4** | **0.8** | **2** | **4** | **12** | **20** | **28** | **36** | **40** |
| **Peak area 1** | 6386 | 64282 | 108714 | 212132 | 506184 | 1074256 | 3145570 | 5132380 | 7173794 | 8998958 | 10624068 |
| **Peak area 2** | 6524 | 64285 | 108468 | 211791 | 506935 | 1058368 | 3144071 | 5128366 | 7150094 | 8956895 | 10606023 |
| **Peak area 3** | 6594 | 64342 | 108666 | 212392 | 505515 | 1057380 | 3142655 | 5125446 | 7161928 | 8954577 | 10604826 |
| **Average peak area** | 6501.3 | 64303.0 | 108616.0 | 212105.0 | 506211.3 | 1063334.7 | 3144098.7 | 5128730.7 | 7161938.7 | 8970143.3 | 10611639.0 |
| **SD** | 105.8 | 33.8 | 130.4 | 301.4 | 710.4 | 9471.0 | 1457.7 | 3481.4 | 11850.0 | 24981.1 | 10780.5 |
| **RSD** | 1.628 | 0.053 | 0.120 | 0.142 | 0.140 | 0.891 | 0.046 | 0.068 | 0.165 | 0.278 | 0.102 |

**Table 2B: Linearity data for methyl paraben**

|  | **Standard concentration (µg/mL)** | | | | | |
| --- | --- | --- | --- | --- | --- | --- |
|  | **0.8** | **2** | **4** | **12** | **28** | **36** |
| **Peak area 1** | 404489 | 916869 | 1826413 | 5207616 | 12704772 | 15930342 |
| **Peak area 2** | 403367 | 902096 | 1819312 | 5199622 | 12700784 | 15995227 |
| **Peak area 3** | 401440 | 896073 | 1810533 | 5180353 | 12638074 | 16038801 |
| **Average peak area** | 259706.0 | 403098.7 | 905012.7 | 1818752.7 | 12681210.0 | 15988123.3 |
| **SD** | 500.7 | 1542.1 | 10700.4 | 7954.8 | 37410.1 | 54577.3 |
| **RSD** | 0.193 | 0.383 | 1.182 | 0.437 | 0.295 | 0.341 |

**Table 3B: Linearity data for butyl paraben**

|  | **Standard concentration (µg/mL)** | | | | | | | | | |
| --- | --- | --- | --- | --- | --- | --- | --- | --- | --- | --- |
|  | **0.02** | **0.2** | **0.4** | **0.8** | **2** | **4** | **12** | **28** | **36** | **40** |
| **Peak area 1** | 16986 | 81161 | 162235 | 199157 | 493802 | 960343 | 2820104 | 6905771 | 8999281 | 9771783 |
| **Peak area 2** | 17366 | 82491 | 159995 | 199403 | 495567 | 958925 | 2819639 | 6917093 | 9000704 | 9782158 |
| **Peak area 3** | 17295 | 83893 | 158666 | 199479 | 494877 | 957946 | 2820301 | 6911677 | 8996810 | 9775275 |
| **Average peak area** | 17215.7 | 82515.0 | 160298.7 | 199346.3 | 494748.7 | 959071.3 | 2820014.7 | 6911513.7 | 8998931.7 | 9776405.3 |
| **SD** | 202.0 | 1366.2 | 1803.8 | 168.3 | 889.5 | 1205.2 | 339.9 | 5662.8 | 1970.4 | 5279.1 |
| **RSD** | 1.174 | 1.656 | 1.125 | 0.084 | 0.180 | 0.126 | 0.012 | 0.082 | 0.022 | 0.054 |
